# Identifying drug substances of screening tool for older persons’ appropriate prescriptions for Japanese

**DOI:** 10.1186/s12877-018-0835-y

**Published:** 2018-07-03

**Authors:** Kaori Nomura, Taro Kojima, Shinya Ishii, Takuto Yonekawa, Masahiro Akishita, Manabu Akazawa

**Affiliations:** 1 Division of Molecular Epidemiology, Jikei University School of Medicine, Tokyo, Japan; 2 Department of Geriatric Medicine, the University of Tokyo, Tokyo, Japan; 3 Public Health and Epidemiology, Meiji Pharmaceutical University, Tokyo, Japan

**Keywords:** ATC, Geriatric patients, STOPP-J, Database, Appropriate prescribing

## Abstract

**Background:**

In 2015, the Japan Geriatric Society (JGS) updated “the Guidelines for Medical Treatment and its Safety in the elderly,” accompanied with the Screening Tool for Older Persons’ Appropriate Prescriptions for Japanese (STOPP-J): “drugs to be prescribed with special caution” and “drugs to consider starting.” The JGS proposed the STOPP-J to contribute to improving prescribing quality; however, each decision should be carefully based on medical knowledge. The STOPP-J shows examples of commonly prescribed drug substances, but not all relevant drugs. This research aimed to identify substances using such coding, as a standardized classification system would support medication monitoring and pharmacoepidemiologic research using such health-related information.

**Methods:**

A voluntary team of three physicians and two pharmacists identified possible approved medicines based on the STOPP-J, and matched certain drug substances to the Anatomical Therapeutic Chemical Classification (ATC) and the Japanese price list as of 2017 February. Injectables and externally used drugs were excluded, except for self-injecting insulin, since the STOPP-J guidelines are intended to cover medicines used chronically for more than one month. Some vaccines are not available in the Japanese price list since they not reimbursed through the national health insurance.

**Results:**

The ATC 5th level was not available for 39 of the 235 identified substances, resulting in their classification at the ATC 4th level. Furthermore, among 26 combinations, 10 products were matched directly to the ATC 5th level of the exact substances, and others were linked to the ATC representing the combination or divided into multiple substances for classification if the combination was not listed in the ATC.

**Conclusion:**

This initial work demonstrates the challenge of matching ATC codes and the Japan standard commodity classification codes corresponding to STOPP-J substances. Since coding facilitates database analysis, the proposed drug list could be applied to research using large databases to examine prescribing patterns in patients older than 75 years or who are frail. Since ATC is not available for some substances, Japanese medicines need the process to be registered in the ATC for an effective screening tool to be developed for STOPP-J.

## Background

Medication prescribing for the elderly is a complex task that requires special care and increased patient monitoring, while appropriate medications are vital for keeping elderly patients healthy, especially those with multiple diseases who use polypharmacy. Japan is known as the most aged country with 26.5% of the population older than 65 [1]. Therefore, a regulatory meeting was established in 2017 to discuss appropriate prescribing and medication use in the elderly and ensure adaptability to the changing medical status of patients [2]. Furthermore, this strategy is expected to reduce the side effects and polypharmacy and ensure reasonable medical costs [2]. Japan has been releasing new drugs to the world, and its regulatory authorities and pharmaceutical companies are expected to provide enough information and formulate precautions for the use of drugs in the elderly. However, since clinical trials mostly exclude the elderly, clinical information for this demographic is scarce for most new drugs, studies are eagerly anticipated, which is true in every country and region.

To aid physicians to prescribe appropriately, the Japan Geriatric Society (JGS) first published guidelines for safe pharmacotherapy in the elderly, and a list of potentially inappropriate medication uses in 2005 [3], which is recognized as the Japanese version of the Beers criteria [4]. It was updated in 2015, providing the Screening Tool for Older Persons’ Appropriate Prescriptions for the Japanese (STOPP-J) for drugs to be prescribed with special caution and drugs to be considered for treatment [5]. Ahead of this, in Europe, the Screening Tool of Older Persons’ potentially inappropriate Prescription (STOPP) [6, 7], proposed by the Ireland study group, is reported as a useful guide for identifying potentially inappropriate medications, particularly for hospital inpatients [8].

Recent evolutions in medical informatics and computerization have enabled researchers to use various databases and analytical tools in their studies. Studies using the databases of regulatory authorities, insurance claims, and medical records, as well as patients’ reports have become popular in public health disciplines and drug development phases recently as well as post-marketing phase. However, there are still challenges associated with the methods of collection, coding, and analysis of data for assessing the accuracy of medication use. The use of identical names or a systematic code for drugs enhances the efficiency of research with large-scale databases [9]. However, coding of medication data depends on the regulatory system, which varies between countries. For example, the National Drug Code in the US (https://www.fda.gov/Drugs/InformationOnDrugs/ucm142438.htm.) differs from the BNF codes (https://data.gov.uk/dataset/176ae264-2484-4afe-a297-d51798eb8228/resource/bac33489-b3dc-47ec-b688-da9cf40e25bd) in the UK. For research on medicine use in the elderly, the AGS Beers criteria [4], STOPP/START [7], and JGS guidelines [5] have been used. However, these guidelines mainly provide drug categories and medication considerations, which lack consistency (Table 1). In addition, they do not specifically define molecular entities, so that researchers have to select drug substances to be studied. Therefore, variation often occurs when using databases to investigate drug use. To facilitate computerized work with databases, Groot et al. [8] proposed a uniform coding for drugs approved in the Netherlands, compliant with the STOPP/START. On the other hand, Japan has several drug-coding systems, depending on the regulatory objective, such as those for labeling information, reimbursement of medical fees, and a third for logistics, but they are solely for domestic use. A variety of proprietary databases is currently available in Japan, and medical terms are mostly compared between them using the International Classification of Diseases (ICD) [10] or the Medical Dictionary for Regulatory Activities (MedDRA) [11], which are internationally recognized in pharmacoepidemiological studies. Unfortunately, due to nationally defined drug coding based on approval, the indications differ, and the classification of medication data may not be consistent across databases. Furthermore, additional efforts for drug identification and matching are necessary when coding systems are different. This process could introduce mismatching and misinterpretation flaws into studies using multiple databases. Therefore, it would be worthwhile to standardize drug codes for international use, e.g., global pharmacovigilance, as the aforementioned Dutch group did by identifying STOPP/START drugs at the substance level and using international coding systems [8]. However, while a local version of the Beers criteria and STOPP/START has been proposed in Japan [12, 13], no guidelines have been presented on how to encode medications. In this paper, we present a proposal to encode drugs in JGS medication guidelines using the Anatomical Therapeutic Chemical Classification System (ATC) [14], supporting the extraction and validity of the medication use data.

**Table 1:** Characteristics of pharmacotherapy criteria for older adults

	AGS Beer’s criteria 2015 ^1)^	STOPP/START v2 2015 ^2)^	JGS STOPP-J 2015 ^3)^
Latest version Developed	AGS	Study group at University College Cork	JGS
Original version (year, developer)	1991, Dr. Beers	2008, Study group at University College Cork	2005, JGS
Target population	United States; 65 and older; Ambulatory, acute, and institutionalized settings	Europe-wide prescribing practices;65 and older; in most clinical settings	Japan; 75 and older; 74 and younger with frailty; 65 and older in need of nursing care; chronic treatment
Therapeutic category / drug	26 potentially inappropriate medication (PIM) 12 PIM due to drug-disease/ drug-syndrome interactions 5 PIM to be used with caution 10 PIM non-anti-infective drug-drug interactions that should be avoided in older adults	80 criteria in STOPP 34 criteria in START	29 groups for drugs to be prescribed with special caution 8 groups for drugs to consider starting
Remarks	Supplement information: “Quality of evidence” and “Strength of Recommendation”	For adverse drug events prevention and cost reduction	Supplement information: “Quality of evidence” and “Strength of Recommendation”

*Abbreviations*:

AGS: American Geriatric Society

JGS: Japan Geriatric Society

PIM: Potentially Inappropriate Medication use in older adults

STOPP: Screening Tool of Older People’s potentially inappropriate Prescriptions

START: Screening Tool to Alert doctors to Right Treatment

STOPP-J: Screening Tool for Older Persons’ Appropriate Prescriptions for Japanese

^1)^American Geriatrics Society. 2015 Beers Criteria Update Expert Panel. American Geriatrics Society 2015 Updated Beers Criteria for Potentially Inappropriate Medication Use in Older Adults. J Am Geriatr Soc. 2015;63:2227–2246

^2)^O’Mahony D, O’Sullivan D, Byrne S, O’Connor M, Ryan C, Gallagher P. STOPP/START criteria for potentially inappropriate prescribing in older people: version 2. Age Ageing. 2015;44:213–218

^3)^Kojima T, Mizukami K, Tomita N, Arai H, Ohrui T, Eto M, et al. Screening Tool for Older Persons’ Appropriate Prescriptions for Japanese: Report of the Japan Geriatrics Society Working Group on“Guidelines for medical treatment and its safety in the elderly.” Geriatrics Gerontology Int. 2016;16:983–1001

## Methods

### Process for listing drug substances

This study was set up voluntarily by three physicians (Akishita, Kojima, and Ishii) and two pharmacists (Akazawa and Nomura) who have experience with studies on drug use in the elderly, using both clinical observations and databases. The STOPP-J 2015 developed by the JGS through systematic review, repeated group discussion, and review by the related academic societies, followed by public consultation [15], was the basis for this study. This JGS tool for medications for older persons does not include details on drug dosage, frequency, or duration of administration, but rather includes drug categories or names. The first step involved drafting a list of approved proprietary names based on JGS guidelines with the support of the Japan Pharmaceutical Information Center (JAPIC), an organization that provides drug information and codes to the Ministry of Health, Labour and Welfare, and pharmaceutical companies. JAPIC provided the English and Japanese names of medicinal substances, supervised by Akazawa and Nomura. The National Health Insurance Drug Price List as of February 2017 [16] was referenced for substance names in Japanese. They included all relevant active substances approved in Japan, which were grouped into specific categories, e.g., the statin category included atorvastatin, simvastatin, pitavastatin, pravastatin, fluvastatin, and rosuvastatin.

In the second step, the physicians, Kojima and Ishii, reviewed the drafted drug names in parallel from the perspectives of clinical treatment of the elderly, supervised by Akishita to reach a consensus. Since the JGS guidelines suggest controlling long-term medication for older persons to avoid untoward systemic adverse events occurring in any case where a drug is used or unused, external drugs and injections were excluded, except for self-injection. Simultaneously, the pharmacists matched the Japanese drug names with the national price list and the English drug names with ATC codes according to pharmacological criteria. Yonekawa helped encoding work. All possible oral indications were considered. Table 2 shows the criteria used to choose substances, categories, and codes.

**Table 2:** Procedures and concepts for listing drugs and codes

Listing drugs	- Listed JGS substances were limited to those approved as medicinal product for oral use in Japan, except insulin. - Listed JGS substances were prescribed for long-term use in general. - If a combination drug is comprised with more than one of listed therapeutic category, combinations were presented in Table 5.
Coding with ATC	- If ATC codes at the 5th level cannot be directly matched to the substance, an alternate 4th level code is proposed by searching online [1]. - The 5th level ATC code is proposed when it is available for the combination, otherwise ATC codes are searched for each substance. - ATC codes for topical use were excluded
Coding with Japan code	- Some vaccines are not available because vaccine is not covered by the pricing list for the national health insurance but by other public support. - The pricing list is available for medicinal products currently in the market.

*Abbreviations:*

JGS: Japan Geriatric Society

ATC: Anatomical Therapeutic Chemical

^1)^WHO Collaborating Centre for Drug Statistics Methodology. ATC/DDD Index 2017. https://www.whocc.no/atc_ddd_index/ Accessed: Accessed 5 June 2017

### Drug classification systems

There are two major global drug classification systems; the Anatomical Therapeutic Classification by the European Pharmaceutical Market Research Association (EPhMRA) [17] and the ATC created by the World Health Organization (WHO) Collaborating Centre for Drug Statistics [14]. Our study used the WHO ATC classification, which has codes at the substance level known as the 5th level.

In Japan, several drug-coding systems exist. The Japan standard commodity classification includes classes for drugs, which appear similar to the 3rd level of the ATC. However, it should be used with caution since it has not been updated since 1990 and, therefore, numerous new drugs are coded as “others.” Based on this system, the National Health Insurance Drug Price List [16, 18] provides 7-digit drug codes at substance level, similar to the ATC 5th level, and 12-digit codes at the product level. In contrast with the ATC process, we extracted the code using the first 7-digit  numeric code from the 12-digit alphanumeric code to represent the substance level.

The selected drugs and the corresponding codes, proposed first by Nomura and Yonekawa, were compared with those formulated separately by the Japanese system vendor, Data Horizon Corporation (https://www.dhorizon.co.jp). Then, the differences were checked and returned to both the authors and the corporation to reach a consensus.

## Results

The drug list is presented in Tables 3, 4, and 5. Of the 236 encoded drug substances, 197 matched the 5th level of the ATC, along with 10 of 26 combinations. No ATC was available at the 5th level for 39 substances and, therefore, they were identified as 4th level substances. If multiple ATCs at the 5th level were available for one substance, the best pharmacological match or the indication-matched ATC was selected and presented with the rest of the possible ATCs. These lists are available as a PDF and spreadsheet at http://docrd.jp/ftp_up/STOPP-J%20List.pdf and http://docrd.jp/ftp_up/STOPP-J%20List.xlsx, and also on the JGS web page for the STOPP-J in Japanese, http://www.jpn-geriat-soc.or.jp/tool/pdf/list_02.pdf and http://www.jpn-geriat-soc.or.jp/tool/xls/list_03.xlsx. Since the JGS’s list was prepared as a support tool for daily medical practices, medicines rarely used or withdrawn were excluded from our list. Medicines used for short-term treatments were also excluded. The STOPP-J shows all Insulin products as drugs to be prescribed with special caution, however, if describing more accurately it recommends prescribers consider to stop sliding scale administration. This indicates that insulins can be prescribed and, therefore, they are excluded from coding.

**Table 3:** Proposal for drug coding of “List of drugs to be prescribed with special caution” ^1)^

Therapeutic category/JAN English name	Japan ^2)^	ATC^3)^
Nervous system: Overall antipsychotic drugs - Antipsychotic drugs		
Aripiprazole Hydrate	1179045	N05AX12
Asenapine Maleate	1179056	N05AH05
Blonanserin	1179048	N05AX
Bromperidol	1179028	N05 AD06
Chlorpromazine Hydrochloride	1171001	N05AA01
Chlorpromazine Phenolphthalinate	1171005	N05AA01
Clocapramine Hydrochloride Hydrate	1179030	N05AX
Clozapine	1179049	N05AH02
Fluphenazine Maleate	1172009	N05AB02
Haloperidol	1179020	N05 AD01
Levomepromazine Maleate	1172014	N05AA02
Mosapramine Hydrochloride	1179035	N05AX10
Nemonapride	1179036	N05AL
Olanzapine	1179044	N05AH03
Oxypertine	1179011	N05AE01
Paliperidone	1179053	N05AX13
Perphenazine	1172006 1172007	N05AB03
Perphenazine Fendizoate	1172004	N05AB03
Perphenazine Maleate	1172013	N05AB03
Perospirone Hydrochloride Hydrate	1179043	N05AX
Pimozide	1179022	N05AG02
Pipamperone Hydrochloride	1179006	N05 AD05
Prochlorperazine Maleate	1172010	N05AB04
Propericiazine (Periciazine)	1172005	N05 AC01
Quetiapine Fumarate	1179042	N05AH04
Risperidone	1179038	N05AX08
Spiperone	1179015	N05 AD
Sulpiride	1179016 2329009	N05AL01
Sultopride Hydrochloride	1179032	N05AL02
Tiapride Hydrochloride	1190004	N05AL03
Timiperone	1179026	N05 AD
Zotepine	1179024	N05AX11
Combination (see Table 5)		
Nervous system: Benzodiazepines		
Alprazolam	1124023	N05BA12
Bromazepam	1124020	N05BA08
Brotizolam	1124009	N05CD09
Chlordiazepoxide	1124028	N05BA02
Clorazepate Dipotassium	1124015	N05BA05
Clotiazepam	1179012	N05BA21
Cloxazolam	1124014	N05BA22
Diazepam	1124017	N05BA01
Estazolam	1124001	N05CD04
Ethyl Loflazepate	1124029	N05BA18
Etizolam	1179025	N05BA19
Fludiazepam	1124019	N05BA17
Flunitrazepam	1124008	N05CD03
Flurazepam Hydrochloride	1124002	N05CD01
Flutazolam	1124024	N05BA
Flutoprazepam	1124027	N05BA
Haloxazolam	1124005	N05CD
Lorazepam	1124022	N05BA06
Lormetazepam	1124010	N05CD06
Medazepam	1124021	N05BA03
Mexazolam	1124025	N05BA
Nimetazepam	1124004	N05BA
Nitrazepam	1124003	N05CD02
Oxazolam	1124013	N05BA
Quazepam	1124030	N05CD10
Rilmazafone Hydrochloride Hydrate	1129006	N05CD
Tofisopam	1124026	N05BA23
Triazolam	1124007	N05CD05
Nervous system: Non-benzodiazepines		
Eszopiclone	1129010	N05CF04
Zolpidem Tartrate	1129009	N05CF02
Zopiclone	1129007	N05CF01
Nervous system: Tricyclic antidepressants		
Amitriptyline Hydrochloride	1179002	N06AA09
Amoxapine	1179001	N06AA17
Clomipramine Hydrochloride	1174002	N06AA04
Dosulepin Hydrochloride	1179027	N06AA16
Imipramine Hydrochloride	1174006	N06AA02
Lofepramine Hydrochloride	1174004	N06AA07
Nortriptyline Hydrochloride	1179004	N06AA10
Trimipramine Maleate	1174005	N06AA06
Nervous system: Selective serotonin reuptake inhibitor (SSRI)		
Escitalopram Oxalate	1179054	N06AB10
Fluvoxamine Maleate	1179039	N06AB08
Paroxetine Hydrochloride Hydrate	1179041	N06AB05
Sertraline Hydrochloride	1179046	N06AB06
Nervous system: Antiparkinsonian drugs – Anticholinergic drugs		
Biperiden Hydrochloride	1162001	N04AA02
Mazaticol Hydrochloride Hydrate	1169004	N04AA10
Piroheptine Hydrochloride	1169003	N04AA
Profenamine Hibenzate	1163002	N04AA05
Profenamine Hydrochloride	1163001	N04AA05
Promethazine Hydrochloride	4413002	R06AD02 D04AA10
Promethazine Hibenzate	4413002	R06AD02 D04AA10
Promethazine Methylenedisalicylate	4413002	R06AD02 D04AA10
Trihexyphenidyl Hydrochloride	1169001 1169002	N04AA01
Systemic hormonal preparations, excl. Sex hormones and insulins: Oral corticosteroids		
Betamethasone	2454004	H02AB01
Cortisone Acetate	2452001	H02AB10
Dexamethasone	2454002	D07AB19 H02AB02
Hydrocortisone	2452002	H02AB09
Methylprednisolone	2456003	H02AB04
Prednisolone	2456001 2456002	H02AB06
Triamcinolone	2454003	H02AB08
Combination (see Table 5)		
Blood and blood forming organs: Antithrombotic drugs		
Acetylsalicylic acid (Aspirin)	1143001 3399007	N02BA01 B01AC06
Apixaban	3339004	B01AF02
Cilostazol	3399002	B01AC23
Clopidogrel Sulfate	3399008	B01AC04
Dabigatran Etexilate Methanesulfonate	3339001	B01AE07
Edoxaban Tosilate Hydrate	3339002	B01AF03
Prasugrel Hydrochloride	3399009	B01AC22
Rivaroxaban	3339003	B01AF01
Ticlopidine Hydrochloride	3399001	B01AC05
Ticagrelor	3399011	B01AC24
Warfarin potassium	3332001	B01AA03
Combination (see Table 5)		
Cardiovascular system: Digitalis		
Digoxin	2113003 2113004	C01AA05
Metildigoxin	2113005	C01AA08
Cardiovascular system: High-ceiling diuretics		
Azosemide	2139008	C03CA
Bumetanide	2139004	C03CA02
Furosemide	2139005	C03CA01
Piretanide	2139007	C03CA03
Torasemide	2139009	C03CA04
Cardiovascular system: Potassium-sparing agents		
Eplerenone	2149045	C03DA04
Spironolactone	2133001	C03DA01
Cardiovascular system: Beta blocking agents		
Alprenolol Hydrochloride	2123002	C07AA01
Arotinolol Hydrochloride	2123014	C07AA
Bufetolol Hydrochloride	2123006	C07AA
Carteolol Hydrochloride	2123005 2149025	C07AA15 S01ED05
Nadolol	2123015	C07AA12
Nipradilol	2149021	C07AA S01ED
Pindolol	2123009 2149011	C07AA03
Propranolol Hydrochloride	2123008 2149014	C07AA05
Cardiovascular system: Alpha1 blocking agents		
Bunazosin Hydrochloride	2149015	C02CA
Doxazosin Mesilate	2149026	C02CA04
Prazosin Hydrochloride	2149002	C02CA01
Terazosin Hydrochloride Hydrate	2149023	G04CA03
Urapidil	2149020	C02CA06
Respiratory system: H_1_ receptor antagonists (1st generation)		
Alimemazine Tartrate	4413003	R06AD01
Chlorpheniramine Maleate	4419001 4419003	R06AB04
Clemastine Fumarate	4419008	R06AA04
Cyproheptadine Hydrochloride Hydrate	4419005	R06AX02
D-chlorpheniramine Maleate	4419002	R06AB04
Diphenhidramine	4411001	R06AA02
Homochlorcyclizine Hydrochloride	4419006	R06AE
Hydroxyzine Hydrochloride	1179005	N05BB01
Hydroxyzine Pamoate	1179019	N05BB01
Promethazine Hibenzate (relisted)	4413002	R06AD02
Promethazine Hydrochloride (relisted)	4413002	R06AD02
Promethazine Methylenedisalicylate (relisted)	4413002	R06AD02
Combination (see Table 5)		
Alimentary tract and metabolism: H_2_ receptor antagonists		
Cimetidine	2325001	A02BA01
Famotidine	2325003	A02BA03
Lafutidine	2325006	A02BA08
Nizatidine	2325005	A02BA04
Ranitidine Hydrochloride	2325002	A02BA02
Roxatidine Acetate Hydrochloride	2325004	A02BA06
Alimentary tract and metabolism: Antiemetic agents		
Metoclopramide	2399004	A03FA01
Promethazine Hibenzate (relisted)	4413002	R06AD02 D04AA10
Promethazine Hydrochloride (relisted)	4413002	R06AD02 D04AA10
Promethazine Methylenedisalicylate (relisted)	4413002	R06AD02 D04AA10
Alimentary tract and metabolism: Drugs for constipation		
Magnesium Oxide	2344002 2344009	A02AA02 A06AD02
Alimentary tract and metabolism: Biguanides		
Buformin Hydrochloride	3962001	A10BA03
Metformin Hydrochloride	3962002	A10BA02
Combination (see Table 5)		
Alimentary tract and metabolism: sulfonylureas		
Acetohexamide	3961001	A10BB31
Chlorpropamide	3961004	A10BB02
Glibenclamide	3961003	A10BB01
Gliclazide	3961007	A10BB09
Glimepiride	3961008	A10BB12
Glyclopyramide	3961002	A10BB
Tolbutamide	3961006	A10BB03
Combination (see Table 5)		
Alimentary tract and metabolism: Alpha glucosidase inhibitors		
Acarbose	3969003	A10BF01
Miglitol	3969009	A10BF02
Voglibose	3969004	A10BF03
Combination (see Table 5)		
Alimentary tract and metabolism: Thiazoridinediones		
Pioglitazone Hydrochloride	3969007	A10BG03
Combination (see Table 5)		
Alimentary tract and metabolism: Sodium-glucose co-transporter 2 (SGLT2) inhibitors		
Canagliflozin Hydrate	3969022	A10BK02
Dapagliflozin Propylene Glycolate Hydrate	3969019	A10BK01
Empagliflozin	3969023	A10BK03
Ipragliflozin L-proline	3969018	A10BK
Luseogliflozin Hydrate	3969020	A10BK
Tofogliflozin Hydrate	3969021	A10BK
Urologicals: Muscarinic receptor antagonists		
Fesoterodine Fumarate	2590015	G04BD11
Imidafenacin	2590013	G04BD
Oxybutynin Hydrochloride	2590005	G04BD04
Propiverine Hydrochloride	2590007	G04BD06
Solifenacin Succinate	2590011	G04BD08
Tolterodine Tartrate	2590012	G04BD07
Musculo-skeletal system: Non-steroidal antiinflammatory drugs (NSAIDs)		
Acemetacin	1145003	M01AB11
Amfenac Sodium Hydrate	1147006	M01AB
Ampiroxicam	1149030	M01 AC
Acetylsalicylic acid (Aspirin) (relisted)	1143001 3399007	N92BA01 B01AC06
Bucolome	1149009	M01AX
Diclofenac Sodium	1147002	M01AB05 S01 BC03 M02AA15
Emorfazone	1148004	N02BG
Etodolac	1149032	M01AB08
Flufenamate Aluminum	1141004	M01AG03
Flurbiprofen	1149011	M01AE09 M02AA19
Ibuprofen	1149001	M01AE01
Indometacin	1145001 1145002	C01EB03 M01AB01 M02AA23
Indomethacin Farnesil	1145005	M01AB01
Lornoxicam	1149036	M01 AC05
Loxoprofen Sodium Hydrate	1149019	M01AE M02AA
Mefenamic Acid	1141005	M01AG01
Meloxicam	1149035	M01 AC06
Mofezolac	1149033	M01AX
Nabumetone	1149027	M01AX01
Naproxen	1149007	M01AE02
Oxaprozin	1149026	M01AE12
Piroxicam	1149017	M01 AC01 M02AA07 S01 BC06
Pranoprofen	1149010	S01 BC09
Proglumetacin Maleate	1145004	M01AB14
Sulindac	1149015	M01AB02
Tiaprofenic Acid	1149025	M01AE11
Tiaramide Hydrochloride	1148001	N02BG
Zaltoprofen	1149029	M01AE
Combination (See Table 5)		

Japanese version available at http://www.jpn-geriat-soc.or.jp/tool/xls/list_03.xlsx or http://www.jpn-geriat-soc.or.jp/tool/pdf/list_02.pdf

ATC: Anatomical Therapeutic Chemical Classification System

JAN: Japanese Accepted Names for Pharmaceuticals

Japan: the first 7-digit numbers of the code of the Japanese drug price list

^1)^Drugs that had been previously approved but do not currently being marketed are excluded. The list includes long-term oral use drugs as a general rule, except self-injection insulin, according to the guidelines (Japan Geriatric Society. Guidelines for Medical Treatment and its Safety in the elderly 2015 (In Japanese). Toyko, Medical View Co., Ltd. 2015)

^2)^A different base adduct may or may not require different codes in Japan; hydroxyzine (1179005 for hydrochloride, 1179019 for pamoate) or promethazines (4413002)

^3)ATC^ codes for topical use were excluded, e.g. A07EA Corticosteroids acting locally, D04AA Antihistamines for topical use; defined daily dose (DDD) are not available for most of those

^4)^The guidelines distinguish sulpiride and sultopride from other antipsychotic drugs

^5)^The guidelines distinguish acetyhlsalicylic acid (aspirine) from other antithrombotic drugs

^6)^The guidelines distinguish oxybutynin from other muscarinic receptor antagonists

^7)^The guidelines does not have metildigoxin (oral), however it is marketed and added to the Table 3 from molecular based perspectives

**Table 4:** Proposal for drug coding of “List of drugs to consider starting” ^1)^

Therapeutic category/JAN English name	Japan ^2)^	ATC ^2)^
Antiparkinson drugs		
Combination (see Table 5)		
Vaccine: Influenza		
Influenza HA Vaccine (A/B)	NA	J07BB02
Adsorbed Influenza Virus Vaccine (H5N1)	NA	J07BB02
Vaccine: Pneumococcal		
Pneumococcal Polysaccharide Conjugate Vaccine(adsorbed)	631140G	J07AL52
Pneumococcus Vaccine	6311400	J07AL01
Cardiovascular system: Angiotensin conversion enzyme (ACE) inhibitor		
Alacepril	2144003	C09AA
Benazepril Hydrochloride	2144007	C09AA07
Captopril	2144001	C09AA01
Cilazapril Hydrate	2144005	C09AA08
Delapril Hydrochloride	2144004	C09AA12
Enalapril Maleate	2144002	C09AA02
Imidapril Hydrochloride	2144008	C09AA16
Lisinopril Hydrate	2144006	C09AA03
Perindopril Erbumine	2144012	C09AA04
Quinapril Hydrochloride	2144010	C09AA06
Temocapril Hydrochloride	2144009	C09AA14
Trandolapril	2144011	C09AA10
Cardiovascular system: Angiotensin receptor blocker (ARB)		
Azilsartan	2149048	C09CA09
Candesartan Cilexetil	2149040	C09CA06
Irbesartan	2149046	C09CA04
Olmesartan Medoxomil	2149044	C09CA08
Telmisartan	2149042	C09CA07
Valsartan	2149041	C09CA03
Losartan Potassium	2149039	C09CA01
Coombination (See Table 5)		
Cardiovascular system: Lipid modifying agents (Statine)		
Atorvastatin Calcium Hydrate	2189015	C10AA05
Fluvastatin Sodium	2189012	C10AA04
Pitavastatin Calcium Hydrate	2189016	C10AA08
Pravastatin Sodium	2189010	C10AA03
Rosuvastatin Calcium	2189017	C10AA07
Simvastatin	2189011	C10AA01
Coombination (See Table 5)		
Urologicals: Drugs for benign prostatic hypertrophy (selective alpha-1 blockers)		
Naftopidil	2590009	G04CA
Silodosin	2590010	G04CA04
Tamsulosin Hydrochloride	2590008	G04CA02
Antineoplastic and immunomodulating agents: Drugs for rheumatoid arthritis		
Actarit	1149031	M01CX
Auranofin	4420001	M01CB03
Bucillamine	4420002	M01CC02
Iguratimod	3999031	M01CX
Leflunomide	3999020	L04AA13
Lobenzarit Sodium	1149020	M01CX
Methotrexate	4222001 3999016	L01BA01 L04AX03
Mizoribine	3999002	L04AX
Salazosulfapyridine	6219001	A07EC01
Tofacitinib Citrate	3999034	L04AA29

ATC: Anatomical Therapeutic Chemical Classification System

JAN: Japanese Accepted Names for Pharmaceuticals

^1)^Drugs that had been previously approved but do not currently being marketed are excluded. The list includes long-term oral use drugs as a general rule, except self-injection insulin, according to the guidelines (Japan Geriatric Society. Guidelines for Medical Treatment and its Safety in the elderly 2015 (In Japanese). Toyko, Medical View Co., Ltd. 2015.)

^2)^the first 7-digit numbers of the code of the Japanese drug price list.A different base adduct may or may not require different codes in Japan; hydroxyzine (1179005 for hydrochloride, 1179019 for pamoate) or promethazines (4413002).

^3)^ATC codes for topical use were excluded, e.g. A07EA Corticosteroids acting locally, D04AA Antihistamines for topical use; defined daily dose (DDD) are not available for most of those.

**Table 5:** Proposal for coding the combination drugs of “List of drugs to be prescribed with special caution” and “List of drugs to consider starting”

Combination drugs (substance name in JAN)	Japan ^1)^	ATC^2)^
“List of drugs to be prescribed with special caution”		
Chlorpromazine Hydrochloride Phenobarbital * Promethazine Hydrochloride	1179100 1179101	R06AD52 promethazine, combinations or N05AA01 chlorpromazine and R06AD02 promethazine
tia Aluminum Glycinate * Magnesium Carbonate *	1143010 3399100	N02BA51 acetylsalicylic acid, combinations excl. Psycholeptics
Acetylsalicylic acid (aspirin) Clopidogrel Sulfate	3399101	N02BA51 acetylsalicylic acid, combinations excl. Psycholeptics or B01AC06 acetylsalicylic acid and B01AC04 clopidogrel
Acetylsalicylic acid (aspirin) Lansoprazole *	3399102	B01AC56 acetylsalicylic acid, combinations with proton pump inhibitors
Metformin Hydrochloride Pioglitazone Hydrochloride	3969100	A10BD05 metformin and pioglitazone
Glimepiride Pioglitazone Hydrochloride	3969101	A10BD06 glimepiride and pioglitazone
Mitiglinide Voglibose	3969102	A10BD Combinations of oral blood glucose lowering drugs or A10BX08 mitiglinide and A10BF03 voglibose
Pioglitazone Alogliptin*	3969103	A10BD09 pioglitazone and alogliptin
Metformin Vildagliptin*	3969104	A10BD08 metformin and vildagliptin
Alogliptin* Metformin	3969105	A10BD13 metformin and alogliptin
“List of drugs to consider starting”		
Entacapone * Carbidopa Hydrate Levodopa	1169102	N04BA03 levodopa, decarboxylase inhibitor and COMT inhibitor
Carbidopa Hydrate * Levodopa	1169101	N04BA02 levodopa and decarboxylase inhibitor
Benserazide Hydrochloride * Levodopa	1169100	N04BA02 levodopa and decarboxylase inhibitor
Azilsartan Amlodipine Besilate	2149121	C09CA09 azilsartan medoxomil and C08CA01 amlodipine
Azelnidipine Olmesartan Medoxomil	2149115	C08C selective calcium channel blockers with mainly vascular effects and C09CA08 olmesartan medoxomil
Amlodipine Besilate Irbesartan	2149118	C09DB05 irbesartan and amlodipine
Amlodipine Besilate Candesartan Cilexetil	2149116	C09DB07 candesartan and amlodipine
Amlodipine Besilate Telmisartan	2149117	C09DB04 telmisartan and amlodipine
Amlodipine Besilate Valsartan	2149114	C09DB01 valsartan and amlodipine
Irbesartan Trichlormethiazide	2149119	C09DA04 irbesartan and diuretics
Candesartan Cilexetil Hydrochlorothiazide	2149111	C09DA06 candesartan and diuretics
Cilnidipine Valsartan	2149120	C08CA14 cilnidipine and C09CA03 valsartan
Termisartan Hydrochlorothiazide	2149113	C09DA07 telmisartan and diuretics
Valsartan Hydrochlorothiazide	2149112	C09DA03 valsartan and diuretics
Hydrochlorothiazide Losartan Potassium	2149110	C09DA01 losartan and diuretics
Atorvastatin Calcium Hydrate Amlodipine Besilate	2190101 2190102 2190103 2109104	C10BX03 atorvastatin and amlodipine

ATC: Anatomical Therapeutic Chemical Classification System

JAN: Japanese Accepted Names for Pharmaceuticals

^1)^The first 7-digit numbers of the code of the Japanese drug price list. The drugs with a different compounding ratio of active substances need different codes in the National Health Insurance Drug Price Standard in Japan. For example, there are bland medicinal products with acetylsalicylic acid 330 mg for anti-inflammatory use (1143010) and 81 mg for antiplatelet (3399100)

^2)^Some combination drugs have individual ATC codes

* Substances are excepted from the STOPP-J list (Table 3and 4)

Combination products were separately listed (Table 5) and were divided into three groups, consisting of one where the combination of exact substances was found in the ATC, such as amlodipine besylate and irbesartan (C09DB05), the ATC representing combination such as levodopa and decarboxylase inhibitor (N04BA02), and another where each substance had an individual ATC. Among the constituent substances in the latter case, the ATCs were presented only for the JGS listed substances. For example, only aspirin was selected from BUFFERIN Combination Tablet® with aspirin, aluminum glycinate, and magnesium carbonate. If the combination product consisted of the same substances in different proportions, different codes were assigned by the price list. For example, combinations of atorvastatin calcium hydrate, and amlodipine besylate were coded 2190101, 2190102, 2190103, or 2109104, depending on their compounding ratio. In addition, drugs can be categorized differently in the Japanese pricing list and have several codes; for example, BUFFERIN Combination Tablet® is 1143010 as “antipyretics, analgesics, and anti-inflammatory agents” and 3399100 as “other agents relating to blood and body fluids” All codes are listed in the tables.

## Discussion

### Identification and application of STOPP-J drug substances

The efforts to reduce inappropriate drug use in elderly patients are likely to have a substantial impact on reducing drug-related morbidity. One major required step is a change in the prescription behavior of physicians, which is influenced by their knowledge and alert systems involving pharmacists, computerized reminders [19], and promotional information from pharmaceutical companies [20]. The current JGS guidelines provide concept and review steps for prescribing to the elderly but do not fully detail specific substances. Thus, our computerized database of standard drug substances, reflecting the STOPP-J with a corresponding coding system, will provide an efficient way to improve physician knowledge about medication for the elderly.

This study revealed that some substances approved in Japan were omitted from the ATC classification system, which was also reported by Groot et al. [8] in reference to the STOPP/START. This may occur when a drug is marketed in Japan only, and the substance or combination is not registered with the WHO Collaborating Center for Drug Statistics. When other countries have the same situation, it would also be necessary to set up the framework to ask the WHO Collaboration Center to include medicinal substances limited to them. This would enhance ATC completeness. To support ATC users, the Uppsala Monitoring Center/WHO Collaborating Centre for International Drug Monitoring does provide the WHO Drug Global with drug information, including Japanese approved drugs and referencing ATC codes at the 5th level, for global pharmacovigilance [21]. Their service supports linking Japanese substances with the ATC, and major global companies use this service for internal databases. It is important to make ATC codes useful in pharmacovigilance and pharmacoepidemiology studies for all Japanese and worldwide drugs and create an official framework to register new substance as soon as possible. This would facilitate drug safety monitoring by pharmaceutical companies and the review of drugs at the class and substance levels. We excluded some medicinal products from the first listing step of the drug indication categories. This paradoxically suggests that researchers run the risk of including appropriately prescribed drugs when extracting data from the drug classification systems.

When the Beers criteria were applied to studies on Japanese elderly patients, hospitalization risk was higher in potentially inappropriate medication users [12] and, in contrast, no association was observed between potentially inappropriate medication use and adverse outcomes [13]. The study using STOPP and START addressed the notion that potentially inappropriate prescribing increased healthcare utilization [22]. Although some drug utilization studies have been reported on the STOPP-J, the future applications of our results to pharmacoepidemiologic clinical studies are worth considering in Japan, similar to a previous study using the Beers criteria in Japan [12, 13]. The use of large databases has become more sophisticated, and 13 Japanese healthcare databases are acknowledged by other entities [23] (e.g., JMDC Claims Database® [24], which provides the names and ATC codes of drugs prescribed from 2005). Some unlisted domestic databases also exist, including the National Database of Health Insurance Claims and Specific Health Checkups of Japan (NDB), which maintain data records from April 2013 provided by the ministry [25]. Currently, no ATC codes are available in the NDB, but the National Health Insurance Drug Price List codes are provided and, therefore, our proposed codes can be used. Another database is the Japanese Adverse Drug Event Report database (JADER), which records spontaneous reports of adverse events to the regulatory agency and lists drug names in plain text, without codes [26]. MID-NET is another prospective database, which was launched in April 2018 by the regulatory agency [27]. It is noteworthy that global comparisons based on the guidelines for medication in the elderly would be complicated or difficult to analyze because substances and their corresponding codes vary.

Currently, there are many therapeutic guidelines and principles for the proper use of medicines, and different definitions are presented worldwide or even in certain countries. Since those guidelines are to be updated periodically in several years, the guidelines propose their philosophies and examples, without identification of drugs. Therefore, interpretation and practice tend to vary by users. When adopting the guidelines, it is important to first define drugs of interest at the component molecule level; however, papers that do not identify the studied drug names might exist. In this research, with reference to the research method of Groot et al. [8] of Ireland, we presented concrete pharmaceutical molecules intended by the STOPP-J proposed by the JGS and proposed corresponding drug codes to be widely used in Japan. The results of this research are expected to be helpful in designing research and validating the actual condition of medical service at a clinical institute. Another important application is to import the drug code list into electronic prescription systems and health information systems so that the system can aid physicians in prescribing cautiously. This application is expected to be used in practice in the near future.

### Limitations of using the list

This study was limited to Japanese drugs for internal use, except insulin, because the JGS guidelines focus on the long-term use of drugs to promote appropriate medications and avoid systemic adverse events in the elderly. The study also excluded drugs mainly used for short-term treatments of less than 1 month, e.g., antipyretics. In addition, based on the JGS, the target population in our list comprised patients older than 75 years who are with or without frailty, which is quite different from other guidelines. The drug list would be useful in research to understand the status of drug prescribing or hypothesize about the trends in total drug use and polypharmacy. However, more information such as dosage regimens and comorbidities is normally required to answer clinical questions. Users also need to consider how to interpret the output. For example, the alerted drug should be able to be monitored or stopped for individual patients. Because the JGS tool is not meant to be a prescription rule, but rather provides information to support physicians’ judgment when prescribing, the dosage regimen and underlying diseases should be mentioned. Lastly, a periodic update of the list is critical for efficient use in practice.

This was the first challenge to identify the STOPP-J substances to be coded. Some difficulties were found through the work in the interpretation of the STOPP-J, for example, insulins, and healthcare data users may misunderstand what the guidelines really proposed. In addition, new medicines need to be timely evaluated to determine whether they should be prescribed with special caution or considered for medication.

## Conclusion

The STOPP-J drug list is proposed in this study as a starting point for discussion for researchers. Our consolidated lists can be used for pharmacoepidemiological database studies. Some WHO ATC codes were omitted owing to regionalized drug availability or combination drugs, which must be considered when using or interpreting the present data.
